# The role of the endolithic alga *Ostreobium* spp. during coral bleaching recovery

**DOI:** 10.1038/s41598-022-07017-6

**Published:** 2022-02-22

**Authors:** Claudia Tatiana Galindo-Martínez, Michele Weber, Viridiana Avila-Magaña, Susana Enríquez, Hiroaki Kitano, Mónica Medina, Roberto Iglesias-Prieto

**Affiliations:** 1 Department of Biology, The Pennsylvania State University, 208 Mueller Lab, University Park, PA 16802 USA; 2 Laboratory of Photobiology, Unidad Académica de Sistemas Arrecifales Puerto Morelos, Instituto de Ciencias del Mar y Limnología, Universidad Nacional Autónoma de México (UNAM), Puerto Morelos, 77580 Cancún, QR Mexico; 3 Okinawa Institute of Science and Technology Graduate School, Okinawa, Japan; 4 The Systems Biology Institute, Shinagawa, Tokyo, Japan; 5 Present Address: Department of Ecology and Evolutionary Biology, University of Colorado, Boulder, USA

**Keywords:** Ecophysiology, Marine biology

## Abstract

In this study, we explore how the Caribbean coral *Orbicella faveolata* recovers after bleaching, using fragments from 13 coral colonies exposed to heat stress (32 °C) for ten days. Biological parameters and coral optical properties were monitored during and after the stress. Increases in both, the excitation pressure over photosystem II (*Qm*) and pigment specific absorption (a*_Chl*a*_) were observed in the stressed corals, associated with reductions in light absorption at the chlorophyll *a *red peak (*D*_*e675*_) and symbiont population density. All coral fragments exposed to heat stress bleached but a fraction of the stressed corals recovered after removing the stress, as indicated by the reductions in *Q*_*m*_ and increases in *D*_*e675*_ and the symbiont population observed. This subsample of the experimentally bleached corals also showed blooms of the endolithic algae *Ostreobium* spp. underneath the tissue. Using a numerical model, we quantified the amount of incident light reflected by the coral, and absorbed by the different pigmented components: symbionts, host-tissue and *Ostreobium* spp. Our study supports the key contribution of *Ostreobium* spp*.* blooms near the skeletal surface, to coral recovery after bleaching by reducing skeleton reflectance. Endolithic blooms can thus significantly alleviate the high light stress that affects the remaining symbionts during the stress or when the coral has achieved the bleached phenotype.

## Introduction

Coral reefs, beside been the most biodiverse marine ecosystems, provide important goods and services to human societies, playing a key role in the local economies of many costal communities^1^. These ecosystems are threatened locally by pollution, overfishing and costal development, and globally by climate change and ocean acidification^2^. Scleractinian corals establish obligate mutualistic symbioses with photosynthetic dinoflagellates^3,4^ in the family Symbiodiniaceae that host in their tissue^5^. The translocation of photosynthates from the algal symbionts provides significant metabolic advantages to the hosts. A clear manifestation of the metabolic advantages associated to the symbiotic nature of reef corals is their rapid calcification rates^6^ that play a critical role in the construction and maintenance of the reef structure itself.

Corals exposed to elevated sea water temperatures, 1.5–2 °C above the summer average, result in dramatic reductions in pigmentation and photosynthetic activity of the dinoflagellates^7–9^, leading to an expulsion of Symbiodinaceae from the coral tissue and a disruption of the mutualistic interaction which reveals the white coloration of the coral skeleton through the transparent animal tissues. This phenomenon, known as coral bleaching, can affect massively large extensions of the coral community^2^. If the heat stress persists, it may result in massive mortality events^10–12^. However, some bleached coral colonies are able to recover pigmentation and functionality^13–15^, though show decreases in fitness. This could result in reef degradation and/or changes in coral community composition^2^, as coral recovery capacity, similar to bleaching vulnerability, differs between species^16^. Recently, increases in coral bleaching events associated with ocean warming have been reported for most coral reefs worldwide^17^. It is expected that massive coral bleaching events will become more frequent and severe, due to climate change^17^, leading to dramatic consequences to reef ecosystems and severe impacts on the goods and services that these ecosystems provide to human societies.

During coral bleaching, reductions in chlorophyl *a* (Chl*a*) mainly due to a decrease in algal symbiont densities^18,19^, result in non-linear increases of the irradiance levels within coral tissue due to the multiple light scattering of solar radiation by the highly reflective coral skeletons^10,11,19^. These increases in the irradiance levels exacerbate light stress during thermal stress episodes, and may result in permanent damage to the photosynthetic algae^18,20,21^. Despite the importance of coral bleaching, very few studies have focused on exploring the mechanisms responsible for the recovery of the coral-algae symbiosis^13,22^. It has been suggested that coral recovery requires a reduction in the excessive light levels *in hospite,* i.e., within the tissue, that affect the remaining symbionts^13,22^.

Previous reports indicate that green fluorescent proteins (GFP)-like pigments may have a potential role in protecting the photosynthetic machinery of the symbiotic dinoflagellates under high light conditions^23,24^. Recently, it was found that during mild heat stress, coral hosts upregulate the production of GFP-like pigments resulting in colorful bleaching events that facilitate coral recovery^22^. In addition to this potential screen provided by GFPs, blooms of the endolithic filamentous algae *Ostreobium* spp. (Chlorophyta: Ulvophyceae: Bryopsidales) within the skeleton have been observed in response to the increase in light availability during coral bleaching^25,26^. *Ostreobium* blooms form distinctive green bands underneath the tissue^26,27^, which present seasonal variations in abundance (i.e., thickness) with maxima in summer. It has been suggested that these endolithic algae and corals may establish a metabolic interaction^25,28,29^. In line with this interpretation, the propagation of *Ostreobium* spp. could help coral recover from bleaching as a potential alternative energy source^25^. However, the increase in *Ostreobium* spp. abundance in the coral skeleton may also provide partial protection to the symbionts against high light stress^13,30^.

To explore the potential role of *Ostreobium* spp. during coral recovery after bleaching, we evaluate the response of *Orbicella faveolata* (Ellis & Solander, 1786) coral fragments to elevated temperature stress. During and after the stress, we monitored the chlorophyll *a* content, dinoflagellate density, pressure over PSII, and coral optical properties. We document that not all coral fragments exhibited a bloom of *Ostreobium* spp. after they bleached. Only those that did present such bloom near the skeletal surface were able to recover once the thermal stress was removed. We conclude that *Ostreobium* blooms facilitate the recovery of the symbiont population in *O. faveolata* after bleaching thanks to its ability to modulate the high local light levels *in hospite* that arise within the bleached/paled coral tissues.

## Results

**Figure 1:**
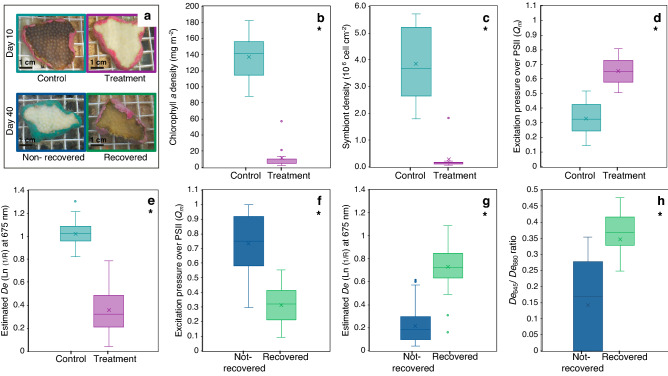
Characterization of the Control and Treatment coral phenotypes**.** Plot (**a**) illustrates with four photographs the differences on day 10 of the experiment between control and treated coral phenotypes, and differences on day 40 between corals that did recover and did not recover after heat stress ceased. The scale bar in photographs is 1 cm. Box plots for comparison of: (**b**) Chlorophyll *a* density (n = 11 fragments per group); (**c**) symbiont density (n = 11 fragments per group); (**d**) excitation pressure over PSII (n = 116 fragments per group); and (**e**) estimated absorbance at 675 nm, *D*_*e675*_ (n = 114 fragments per group), between control (light blue) and heat treated (pink) *Orbicella faveolata* fragments, at the end of heat stress treatment (day 10); Comparation of the (**f**) excitation pressure over PSII (n = 25 fragments per group); and (**g**) estimated absorbance at 675 nm (n = 114 spectra per group), between coral colonies that recovered (green) and did not recover (blue), at the end of the recovery period (day 40); Comparation of the (**h**) 645/680 ratio of the second-derivative analysis of the absorption spectrum (day 10) between coral colonies that recovered (green) and did not recover (blue) (n = 342 spectra per group). Boxes encompass the 25 and 75% quartiles of the variation. The central line corresponds to the median, and bars extend to the 95 and 5% of the confidence limits. Asterisk denotes significant differences among coral groups (T-test, p < 0.05).

All coral fragments exposed for 10 days to heat stress (32 °C) showed a significant reduction in pigmentation (11.93 ± 15.95 mg Chl*a* m^−2^, mean ± SD) with respect to the controls (135.60 ± 32.83 mg Chl*a* m^−2^) (*t*_(13)_ = 10.79, p < 0.01) (Fig. 1a,b; Supplementary Table S1). This was mainly due to a 93% reduction in the symbiotic algal population (*t*_(12)_ = 8.03, p < 0.01) (Fig. 1c) with 10% variation in the Chl*a* content per cell (Supplementary Fig. S2; Supplementary Table S1). Corals exposed to elevated temperature showed two times higher excitation pressure over photosystem II (*Q*_*m*_) (0.65 ± 0.08) than controls (0.32 ± 0.10) (*t*_(223)_ = − 25.25, p < 0.01) (Fig. 1d; Supplementary Table S1). After returning the temperature to control levels (28 °C), some coral fragments recovered in both pigment content and symbiont cell density (Fig. 1a) and exhibited a significant reduction of *Q*_*m*_ (0.32 ± 0.12) at the end of the recovery period (40 days, Fig. 1f; Supplementary Table S2) to return *Q*_*m*_ values of controls. Another group of thermally-stressed coral fragments, however, never recovered and maintained higher *Q*_*m*_ values (0.68 ± 0.23) (*t*_(37)_ = 6.67, p < 0.01) (Fig. 1f; Supplementary Table S2). Supplementary Table S4 indicate the number of coral fragments that recover or not at the end of the experiment for each colony studied.

### Optical characterizations

The significant reduction in coral pigmentation of the stressed coral fragments (after 10 days of heat treatment) was also expressed in a significant reduction in absorbance at 675 nm (*D*_*e675*_ = 0.36 ± 0.18) relative to controls (*D*_*e675*_ = 1.02 ± 0.08) (*t*_(168)_ = 36.12, p < 0.01) (Fig. 1e; Supplementary Fig. S1; Supplementary Table S1). After removing the stress, recovered corals showed significant increases in *D*_*e675*_ (0.72 ± 0.20) relative to non-recovered corals (0.22 ± 0.15) (*t*_(49)_ = − 11.07, p < 0.01) (Fig. 1g; Supplementary Table S2). Absorbance values (*D*_*e675*_) of the recovered coral fragments, however, were still significantly smaller 30 days after reducing temperature to 28 °C than the values determined for control corals (*t*_(31)_ = − 7.40, p < 0.01) (Fig. 1e,g; Supplementary Table S2). At the end of the recovery period, corals that did not recover showed significantly lower *D*_*e675*_ (0.21 ± 0.15) than those registered during the last day of heat stress (*t*_(150)_ = 4.41, p < 0.01; Fig. 1e,g, Supplementary Table S2).

**Figure 2:**
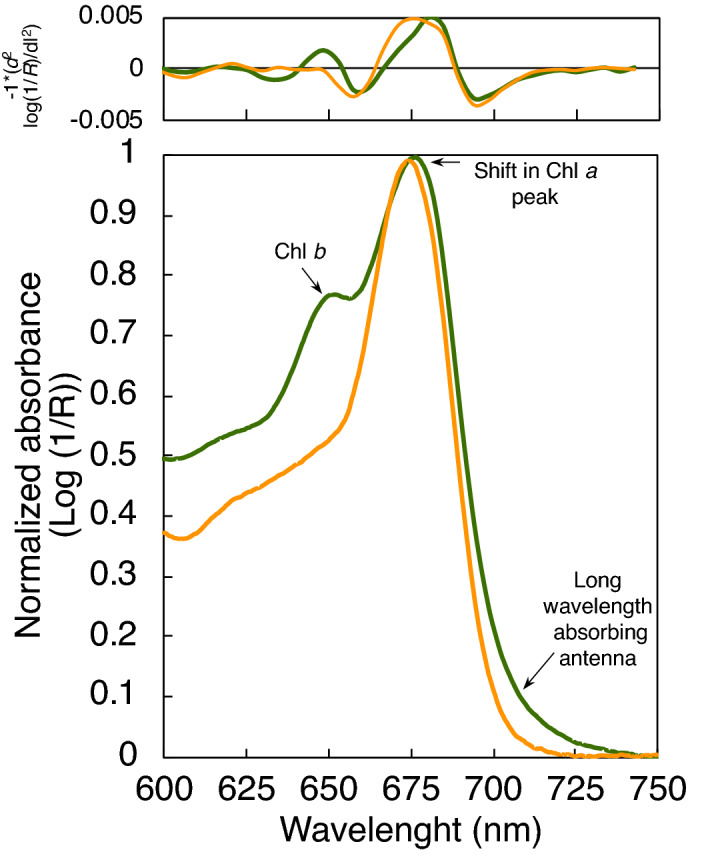
Spectroscopic optical properties of *Orbicella faveolata* and *Ostreobium* spp. Comparison of the estimated in vivo light absorption spectra of the coral *Orbicella faveolata* (orange solid line) and that of the chlorophyte endolithic algae *Ostreobium* spp. (green solid line) living within the coral skeleton of this species. The spectrum of *Ostreobium* spp. was obtained after removing the coral tissue with water and pressurized air. Absorption spectra were normalized to the maximum peak of absorption of Chl*a* in the red band of the spectrum. Top panel represents second derivative analysis of the absorption spectra.

**Figure 3:**
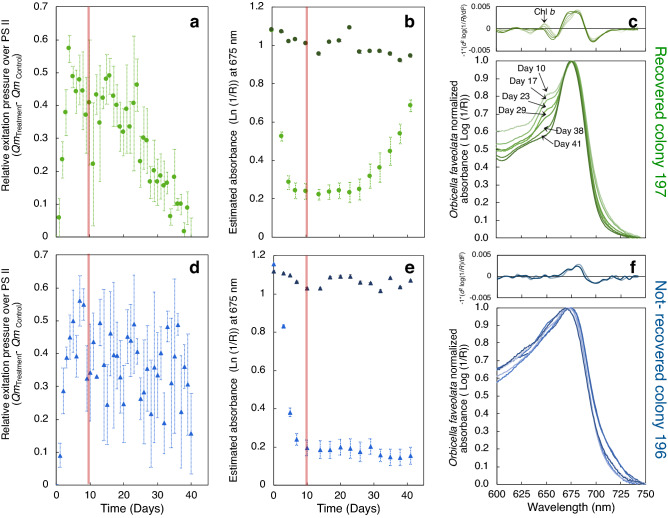
Variation in the physiological parameters and spectroscopic optical properties of *Orbicella faveolata* coral fragments, during the bleaching and recovery experiment (B-Re). Plots (**a**,**d**) illustrate changes (Average ± SE) in the relative excitation pressure over photosystem II (Δ*Q*_*m*_ = *Q*_*m treatment*_* − Q*_*m control*_) in: (**a**) colony 197 fragments (n = 37); and (**d**) colony 196 fragments (n = 37). Red vertical solid line represents the end of the heat stress period. Plots (**b**,**e**) compare the estimated absorbance (*D*_*e*_ = Ln (1/*R*)) at 675 nm (*D*_*e675*_, Average ± SE), during the B-Re experiment, in: (**b**) colony 197 fragments; and (**e**) colony 196 fragments. Light colored symbols represent heat-stressed corals (n = 18), and dark colored symbols represent control corals (n = 19). Red vertical solid line indicates the end of the heat stress period. Plots (**c**,**f**) describe the variation in the average absorption spectra of: (**c**) colony 197 fragments; and (**f**) colony 196 fragments; on the last day of the heat stress treatment (32 °C, day 10, light green line and light blue line respectively), and during the recovery period (28 °C, days 11 to 41, dark green line and dark blue line respectively). Spectra were normalized to the maximum peak of absorption of Chl*a*. Top panels represent the second-derivative analysis of the light absorption spectrum.

We also observed that the corals that recovered had a characteristic absorption band at 645 nm at the end of the heat stress treatment (Fig. 3c), and a significantly higher D_*e645*_/D_*e680*_ ratio on the second-derivative analysis of their absorption spectra (0.35 ± 0.12 and 0.14 ± 0.15 for recovered and not-recovered corals respectively) (*t*_(20)_ = − 3.73, p < 0.01) (Fig. 1h; Supplementary Fig. S1; Supplementary Table S1). The absorption band at 645 nm is consistent with the absorption peak of chlorophyll *b* (Chl*b*), a pigment only present in *Ostreobium* spp., while the absorption peak at 680 nm points to a characteristic shift in the maximum absorption peak of Chl*a* of *Ostreobium* spp.^31,32^, relative to the 675 nm Chl*a* absorption peak that characterizes the light absorption spectra of the symbiotic algae (Fig. 2).

### Comparative analysis between recovered and not-recovered bleached corals

To better understand coral bleaching recovery, we analyzed in more detail the optical properties of coral fragments from two different colonies exposed to the same heat stress treatment, hereafter coral colony 197 and coral colony 196. Genotypic analysis indicates that fragments from both coral colonies belonged to the same animal genotype (Orb 1512) and also contained the same dominant algal symbiont species, *Cladocopium* ‘C7’ (Supplementary Table S4). Despite these similarities, coral fragments showed a contrasting response after removing heat stress, only fragments from colony 197 recovered (Fig. 3).

Similarly to the results previously described, during the heat stress period fragments from both coral colonies showed significant increases in *Q*_*m*_ with their respective colony control: 0.26 ± 0.06 and 0.61 ± 0.24 in control corals and treatment corals respectively for colony 196 (*t*_(5)_ = 3.36, p = 0.01), and 0.21 ± 0.09 and 0.62 ± 0.35 in control corals and treatment corals respectively in colony 197 (*t*_(5)_ = 2.79, p = 0.01; Supplementary Table S3). Only fragments from colony 197 showed a reduction in *Q*_*m*_ once temperature returned to normal, 28 °C (0.20 ± 0.12, *t*_(3)_ = − 2.47, p = 0.05) (Fig. 3a,d; Supplementary Table S3). In both coral colonies fragments, increases in *Q*_*m*_ during the experimental heat stress period resulted in a reduction in the light absorption cross section, which was detected as changes in *D*_*e675*_ (1.04 ± 0.07 and 0.20 ± 0.08 in control and treatment corals respectively for colony 196 (*t*_(19)_ = − 23.33, p < 0.01); and 1.01 ± 0.05 and 0.25 ± 0.11 for colony 197 (*t*_(19)_ = − 21.32, p < 0.01)) (Fig. 3b,e; Supplementary Table S3). Thirty days after temperature returned to 28 °C, coral fragments of colony 197 showed a significant increase in *D*_*e675*_ (0.71 ± 0.15; *t*_(14)_ = − 8.02, p < 0.01) (Fig. 3b; Supplementary Table S3).

### Spectroscopic analysis

Spectroscopic analyses along the 600–750 nm range of the in vivo light absorption spectra, revealed that after ten days under heat stress, coral fragments from both colonies showed a similar absorption band at 675 nm relative to controls (Fig. 3c,f). Coral fragments from colony 197 also showed a characteristic shoulder around 650 nm. During the stress period, increases in absorbance at 645 nm (*D*_*645*_) were observed for those fragments from colony 197, reaching a maximum on day 10 with a posterior reduction during the recovery period (Supplementary Fig. S3a). Second-derivative analyses of these light absorption spectra showed: (i) a characteristic shoulder at 645 nm consistent with the presence of Chl*b* (Supplementary Fig. S4a); and (ii) a shift in the Chl*a* peak of absorption in the red band from 675 to 680 nm (Fig. 3c; Supplementary Fig. S3b). Both findings are indicative of the presence of *Ostreobium* spp. near the surface of the skeleton. Increases in *D*_*e645*_ showed a linear relationship (R^2^ = 0.42, p < 0.05) with the maximum peak of absorption of Chl*a* in the red band (Supplementary Fig. S3c). In contrast to these results, second-derivative analysis of the absorption spectra of colony 196 fragments did not show any absorption peak at 645 nm (Fig. 3f), supporting that *Ostreobium* spp. did not bloom near the surface of the coral skeleton, and thus, was not detectable by the spectroscopic analysis.

**Figure 4:**
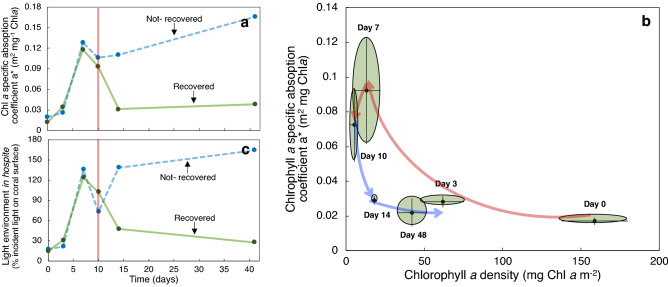
Variation of the chlorophyll *a* specific absorption coefficient (a*_Chl*a*_; m^2^ mg Chl*a*^−1^) and the predicted light environment *in hospite* of *Orbicella faveolata* fragments, during the B-Re experiment. Plots (**a**,**c**) describe the variation in: (**a**) the chlorophyll *a* specific absorption coefficient (a*_Chl*a*_); and (**c**) the predicted light environment in hospite of the symbiotic algae through time in a recovered colony (green solid line) and in a colony that did not recover after the heat stress treatment (blue dashed line). Red vertical solid line represents the end of the heat stress period. Plot (**b**) describes the variation of a*_Chl*a*_ as a function of changes in Chl*a* density in the intact coral. Day 1 to day 10 correspond to the heat stress period (32 °C, red arrows) whereas day 11 to day 40 represent the corals during the recovery period (28 °C, blue arrows).

In coral fragments from colony 197, the *Ostreobium* spp. bloom detected coincided with a drastic reduction of the Chl*a* specific absorption coefficient (a*_Chl*a*_) determined for the same fragments by the last day of heat stress treatment, and that it was maintained during the recovery period (Fig. 4a,b). After reducing temperature to 28 °C, a reduction in *D*_*e645*_ was observed along with a shift in the Chl*a* peak from 680 to 675 nm (Supplementary Fig. S3), suggesting that the recovery of the symbiotic algal population within the coral tissue masked the *Ostreobium* spp. bloom in the skeleton.

### Numerical model

**Figure 5:**
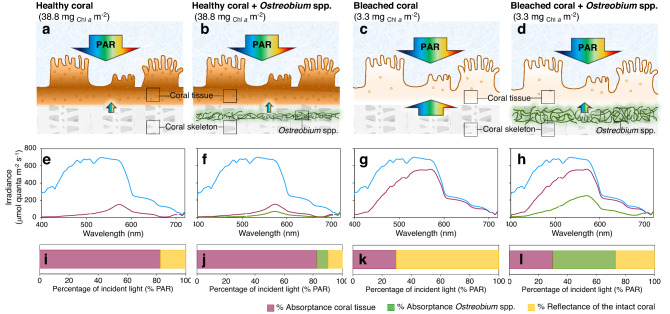
Numerical model for light transmission in corals. Light transmission simulation for a coral at 5 m depth was calculated for: (**a**) a healthy phenotype (38.8 mg _Chl *a*_ m^−2^) with a white coral skeleton; (**b**) a healthy phenotype (38.8 mg _Chl *a*_ m^−2^) with a coral skeleton colonized by the endolithic algae *Ostreobium* spp.; (**c**) a bleached phenotype (3.3 mg _Chl *a*_ m^−2^) with a white coral skeleton; and (**d**) a bleached phenotype (3.3 mg _Chl *a*_ m^−2^) with a coral skeleton colonized by *Ostreobium* spp. Plots (**e–h**) describe changes in the light absorption spectra along the PAR range (400–720 nm) after penetrating 5 m depth into the water column (light blue solid line), and after crossing different layers into the coral structure, which comprises: the coral tissue layer (pink solid line), and the *Ostreobium* spp. algae layer located 0.1 mm below the coral tissue (green solid line); under different scenarios: plots (**e**,**f**) represent a healthy coral with out and with *Ostreobium* spp. in the coral skeleton surface; plots (**g**,**h)** represent a bleached coral with out and with a coral skeleton colonized by *Ostreobium* spp. Finally, plots (**i**–**l**) describe the percentage of PAR utilized by each layer inside coral: coral tissue (pink), *Ostreobium* spp. (green) and coral reflectance (yellow), under the 4 former scenarios.

The numerical model describes the variation in light transmission in corals as Chl*a* density changes, it also calculates the fraction of the incident light that *Ostreobium* spp. are able to absorb when they are located at different depths in the skeleton. Additionally, it describes changes in the optical properties of the skeleton due to the *Ostreobium* spp. bloom, and finally, it calculates the light environment *in hospite* for the symbionts as *E*_*o*_ = (1 + *2R*) *E*_*d*_, where *R* is the coral skeleton reflectance, *E*_*d*_ is the incident downwelling irradiance on the coral skeleton and 2 is the amplification factor for a flat surface^10^ (Fig. 5).

**Figure 6:**
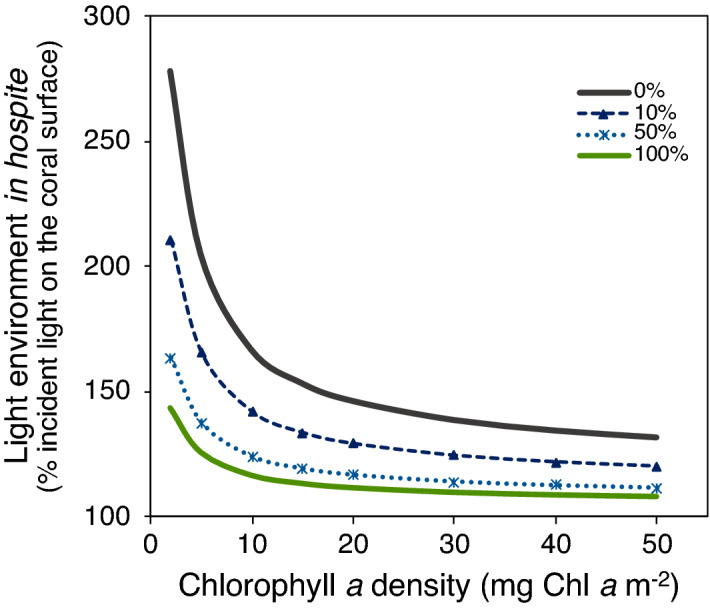
Variation in the light environment in hospite. The predicted variation of the light environment in hospite by the numerical model is calculated for corals at 5 m depth as: *E*_*o*_ = (1 + *2R*) *E*_*d*_, where E_d_ is the incident downwelling irradiance into the coral skeleton; and 2 is the amplification factor for a flat scattering surface. Due to the complex microstructure of coral skeletons, this amplification factor can be much larger (Enríquez et al. 2017) resulting in a larger amplification of the internal light environment. Plot (**a**) describes predicted changes in the in hospite light environment of the symbionts within intact coral fragments, as a function of changes in Chl*a* density and with different presence of *Ostreobium* spp. (abundance) on the surface of the coral skeleton: 100% (green solid line), 50% (light blue dotted line), 10% (dark blue dashed line) or when it is absent in the coral skeleton (gray solid line).

The results of the numerical model indicated that the irradiance available for *Ostreobium* spp. in the coral skeleton increases with reductions in coral pigment density. The tissue of a healthy coral is able to absorb 82.3% of the incident irradiance in the PAR range. When *Ostreobium* spp. are present in the skeleton it is able to absorb up to 7.8% of the incident light in the coral. (Fig. 5i,j). In contrast, tissues of bleached corals only absorb 29.8% of the incident irradiance, but when *Ostreobium* spp. blooms are present, they are able to absorb up to 43% of the incident light (Fig. 5k,l). The numerical model also revealed that the presence of *Ostreobium* spp. near the surface of the coral skeleton affects its optical properties by reducing coral reflectance to 29% (Fig. 5l). However, the capacity of *Ostreobium* spp. to reduce coral skeleton reflectance declines exponentially with both: their position in the coral skeleton and the variation in *Ostreobium* spp. abundance. When *Ostreobium* spp. are located deeper than 2.5 mm in the skeleton, their contribution to modify skeleton reflectance is negligible (Supplementary Fig. S5a). However, when they are located near the surface of the skeleton, even in small density, *Ostreobium* spp. are still able to reduce the reflectance of coral skeletons and, in consequence, the light environment of the symbionts *in hospite* (Fig. 6, Supplementary Fig. S5b).

## Discussion

*Orbicella faveolata* exposed experimentally for 10 days to thermal stress (32 °C) showed significant reductions in pigmentation due, primarily, to reductions in symbiont algal density (Fig. 1a–c; Supplementary Fig. S2). These results coincide with those previously reported during natural bleaching events^7,13,14^ and coral bleaching experiments^7,9,19,22,33^. Bleached coral fragments showed a significant increase in the maximum excitation pressure over photosystem II, *Q*_*m*_*,* relative to controls (Fig. 1d). After removing heat stress, only some stressed coral fragments presented signs of recovery, as reflected by the increases measured in the peak of light absorption at 675 nm, *D*_*e675*_ (Fig. 1g). Second derivative analyses of the light absorption spectra allowed detection of an absorbance peak at 645 nm consistent with the presence of Chl*b* from *Ostreobium* spp. After ten days of exposure to heat stress, only corals that were able to recover, presented the absorbance peak at 645 nm. A more detailed analysis applied to coral fragments derived from two colonies genetically identical that contained the same dominant algal symbiont (Fig. 3; Supplementary Table S4), showed that the presence of large populations of *Ostreobium* spp. near the surface of the skeleton was the only identifiable difference between the fragments that were able to recover after the stress and those that were not. Our findings thus suggest, that the presence of *Ostreobium* spp. on the skeleton facilitates coral recovery after bleaching.

*Ostreobium* spp. is a filamentous chlorophyte that forms green bands within the skeleton of most coral species and most calcareous rocks in reef environments^27,34–36^. Green bands dominated by *Ostreobium* are observed in skeletons of massive coral colonies with slow growth^27^ compared to branching coral colonies where no green bands are observed. The fast growth of branching corals induces a decrease of microborer abundance toward apexes^36,37^ and therefore the absence of green bands. As a chlorophyte, the photosynthetic apparatus of *Ostreobium* spp. contains both chlorophyll *a* and *b*^31,32^. The in vivo spectra also indicate the presence of a long wavelength absorbing antenna forming a distinctive shoulder in the absorption spectrum at around 710 nm^32^. In healthy corals, the amount of light that can penetrate the skeleton represents less than 1% of the incident radiation^38^*,* supporting that *Ostreobium* spp. are adapted to live under extremely low light levels within the coral skeleton maintaining, under those conditions, low metabolic rates^28,39^. However, during thermal stress, when the coral experiences significant reductions in symbiont density, before and after reaching the coral bleaching phenotype^9,19^, multiple scattering of solar radiation is significantly amplified and light availability within the skeleton increases^10–12^, which allow *Ostreobium* spp. to bloom and increase its presence near the surface of the skeleton^25,40^. This bloom of *Ostreobium* spp. may be involved in other additional processes^41^, such as nutritional exchanges^25,29,42^, coral skeletal bioerosion^39^ and/or coral recovery^13^. However, its potential contribution in coral recovery has not been sufficiently investigated^13^.

Due to its high reflectivity, coral skeletons function as a secondary light source for symbionts in hospite, increasing the internal light environment^10^. Thus, the total flux absorbed by the symbionts in front of a flat scattering surface (Φ_abs_) can be calculated as Φ_abs_ = Φ^(i)^_abs_ + Φ^(s)^_abs_ = (1 + 2*R*) Φ^(i)^_abs_, where *R* is the reflectance of the surface, Φ^(i)^_abs_ is the radiant flux absorbed from the incident beam, and Φ^(s)^_abs_ is the flux absorbed by the particle from the light backscattered by the surface^10^. This shows that a non-absorbing surface (R = 1) can enhance the absorption of the algae by a factor of three. However, due to the complex morphologies of coral skeletons, this factor can be larger for a symbiont in hospite^10–12,43^. These characteristics render corals as one of the most efficient light collectors in nature, which is fundamental under optimal conditions. This efficiency comes with a trade-off as during severe levels of thermal stress and under coral bleaching it exposes the remaining symbionts to extraordinary high light stress levels. Therefore, the capacity of the symbionts to recover could be enhanced by: (i) reducing the reflectivity of the coral skeleton^13^ and/or (ii) the upregulation of GFP like pigments in the coral tissue that can function as optical dampers^22^ (Fig. 6).

Two main scenarios have been proposed to explain the effects of multiple light scattering by the coral tissue and its skeleton in modulating the light environment of the symbionts in hospite^10,11,33,43–46^. The first one assumes that the coral tissue is practically transparent, and the coral skeleton is a highly scattering structure that diffuses light isotropically increasing the optical pathlength^10,11^. In contrast, the second scenario proposes that only near infrared (NIR) irradiance levels in corals are determined by the optical properties of the skeleton, while irradiance levels in the visible range are controlled by the scattering properties of the coral tissue itself^43,45^. If the former scenario is correct for all coral species, during coral bleaching, coral tissue will become white after losing its symbionts. However, our observations showed that when *O. faveolata* bleaches, the tissue becomes transparent revealing a clear view of the skeleton through the coral tissue (Fig. 1e) suggesting that in this species, coral tissue has a negligible effect in the modulation of the internal light field.

Accordingly, in *O. faveolata* the light field in hospite is primarily determined by the incident irradiance, coral pigmentation and distribution, and the reflectance of the skeleton. Our numerical model highlights how reductions in coral pigmentation (i.e., Chl*a* density) increase the amount of incident irradiance reaching the skeleton, which results in significant amplification of the light environment in hospite for the algal symbionts due to the particular scattering properties of the white coral skeleton^10–12,44^ (Fig. 6). During the thermal stress treatment, both coral colonies showed significant increases in the light environment *in hospite* of the symbionts (Fig. 4c), depicted by the large increases in the specific light absorption, a*_Chl*a*_, by the remaining symbionts (Fig. 4a,b). In colony 196 fragments (not-recovered), such high light *in hospite* environment was maintained during the whole recovery period (Fig. 4c). This condition positioned the remaining symbionts into a positive feedback loop^12^, where excessive irradiance impedes its recovery. In contrast, in the fragments from colony 197 the reduction in skeleton reflectance derived from the increasing presence of *Ostreobium* spp. (Fig. 5l), significantly mitigates the light environment of the symbionts *in hospite* during the recovery period (Fig. 5c). This reduction could be quantified by the variation in a*_Chl*a*_ (Fig. 5a,b) or a*_sym_ of the remaining symbionts (not estimated in this study but see Refs.^9,19^). Increases in light absorption (*D*_*e675*_) and reductions in the PSII excitation pressure, *Q*_*m*_*,* during the recovery period in colony 197 fragments (Fig. 3a,b), indicate that the *Ostreobium* spp. bloom favored the recovery of the symbionts *in hospite* by removing the excess of irradiance and reducing light stress.

Due to potential reductions in coral calcification rates during the application of the thermal stress treatment and after bleaching, and due to the differences among organisms in the rate of recovery of the initial pigmentation, it is difficult to predict the exact time that the *Ostreobium* spp. bloom can remain near the surface of the skeleton. However, during this time, its effect on the reduction in skeleton reflectance and thus the light environment *in hospite* of the symbionts will be maintained (Fig. 6). Accordingly, pigment absorption efficiency of the symbionts will be reduced (Fig. 4b), leading to a less efficient coral for harvesting light, although more robust against future stressful conditions that result in the exacerbation of light stress for the symbionts. This interpretation could explain why during and after massive bleaching events some coral colonies or areas of a particular colony are able to tolerate same levels of heat stress without presenting any sign of bleaching, than other colonies or areas within the same colony with the same symbiont composition, as documented in previous studies^47^.

The spectroscopic analyses performed in this study evidenced that only a subsample of the coral fragments exposed to heat stress presented an *Ostreobium* spp. bloom in their skeleton. This differential response could be due to (i) the photo acclimatory capabilities of *Ostreobium* spp., (ii) drastic changes in the light environment *in hospite* during the heat stress period; and/or (iii) the absence of *Ostreobium* spp. in the coral skeleton before heat stress. Previous work has demonstrated that endolithic algae in the coral *Montipora monasteriata* has the capacity to acclimate to increased irradiances during a bleaching event^40^. However, a combined thermal and light stress could cause a major damage on the endolithic algae^40^. In our experiments, fast reductions in symbiont density during heat stress could produce a sudden increase of light levels in coral skeletons, causing excessive photodamage on *Ostreobium* spp. and in consequence inhibiting its bloom near the surface of the skeleton. Currently, more that 120 *Ostreobium* taxonomic units (near species level) have been discovered in coral skeletons^48,49^. Each taxonomic unit may display a different physiology^50^ and thus, a different abundance in the skeleton. All these differences could explain the differential coral skeleton colonization by these endolithic algae, as well as their contrasting contribution in the recovery pattern of corals. Further investigation needs to be done for a complete understanding of this contribution.

Our spectroscopic characterization also showed that the presence of a long wavelength absorbing Chl*a* allows *Ostreobium* spp. to collect light in a spectral range above 700 nm that is not utilized neither by the animal host nor the symbiont *in hospite* (Fig. 5f,h). This *Ostreobium*’s antenna represents an advantage for a photosynthetic organism growing in a dim environment, which is shaded principally by the light absorption of the pigmented coral tissue. However, light absorption by the skeleton also limits the depth range in which light penetrates the coral skeleton*.* When *Ostreobium* spp. is deeper than 3 mm in the coral skeleton the amount of light that can be absorbed is negligible in all wavelengths (Supplementary Fig. S5). At such depths the presence of the long wavelength antenna does not provide any advantage to *Ostreobium* spp. Similarly, depth-dependent changes in the spectra composition of light also limits the utility of the long wavelength antenna of *Ostreobium* spp. to the shallowest parts of the reef.

The coral bleaching recovery mechanism previously described^22^ and the mechanism proposed herein highlights the importance of the processes that can contribute to reduce the light stress experienced by the symbiotic algae *in hospite*. These mechanisms can be fundamental to facilitate the recovery of the surviving symbionts after bleaching and are not mutually exclusive. They may represent different coral strategies adopted to prevent excessive increases in the light environment of the symbionts *in hospite*, either by reducing the light scatter of the coral tissue through the GFP like pigments upregulation^22^, or/and by reducing the reflectance of the coral skeleton. More work is still needed to understand both mechanisms within the context of coral bleaching recovery.

## Materials and methods

### Coral collection

Thirteen *Orbicella faveolata* coral colonies were sampled at 8 and 10 m depth at Puerto Morelos, Quintana Roo, Mexico under the collection permit granted by the Mexican government: No.DGOPA.08696.251011.3021. One fragment (25–30 cm^2^) from each coral colony was collected and transported in a black bag inside a cooler to the mesocosm system at the Unidad Académica de Sistemas Arrecifales (Universidad Nacional Autónoma de México, UNAM). Corals were cut into small 3–5 cm^2^ fragments and maintained in an aquarium system at 28 °C for their acclimation and recovery after the sampling and manipulation. The aquarium system consisted of 2 semi-closed 150 L tanks (Control and Treatment) of running seawater from the reef lagoon, located outdoors under natural sunlight illumination. An average water flow of 0.3 L min^−1^ and a turnover rate of 8 h was used to maintain the experimental corals under optimal water conditions. Irradiance levels in tanks were also maintained at values similar to those at the collection site using neutral density filters (average exposure of 9 mol quanta m^2^ day^−1^). Water temperature was controlled using an arrangement of heaters (Process Technology, USA, model TA1.8117-P1) and titanium chillers (Artica^®^, model DBA-075) that allowed maintenance under stable conditions.

### Bleaching and recovery experiments (B-Re)

Bleaching and recovery experiments (B-Re) were performed modifying the methodology described by Scheufen et al.^9^. Briefly, coral fragments of each colony were separated equally into Control and Treatment tanks (Supplementary Table S4). Corals were acclimated to the aquarium conditions (7 days) with a temperature maintained at 28 °C (± 0.5 °C) before beginning the B-Re experiments. At night during day 0, water temperature in the treatment tank was increased overnight to 32 °C and coral fragments were exposed to this temperature level (32 ± 0.4 °C) for 10 days (experimental period of the heat stress treatment). By the night of the 10th day, water temperature was reduced back to 28 °C and maintained stable for the next 30 days (recovery period). Coral fragments in the control tank were exposed to 28 ± 0.5 °C during both, experimental and recovery, periods. Water temperature and photosynthetic active radiation (PAR) were recorded every 5 min on the aquaria using a temperature data logger (HOBO Pendant^®^, ONSET), and an underwater quantum sensor (LI-192, LI-COR^®^) attached to a light sensor logger (LI-1500, LI-COR^®^) respectively. Excitation pressure (*Q*_*m*_) over photosystem II (*PSII*), coral pigment content and coral reflectance, were monitored before (Supplementary Fig. S1), during and after thermal stress in both, control and treatment corals. For genetic analysis on the Symbiodiniaceae and coral host (see Supplementary materials), and chlorophyll *a* determinations, coral samples from each colony were flash-frozen at the end of the day for every time point (days 0, 3, 7, 10, 14, 17, and 41).

### Excitation pressure over PSII (Q_m_)

To determine *Q*_*m*_, both effective *(∆F/Fm′*) and maximum quantum yield (*Fv*/*Fm*) of *PSII* were measured daily on each coral fragment at noon and 15 min after dusk respectively using an underwater fluorometer (diving PAM, WALZ). *Q*_*m*_ was calculated according to Iglesias-Prieto et al.^51^ using the equation *Q*_*m*_ = 1 − [(*∆F∕Fm*_at noon_)*/*(*Fv/Fm*_at dusk_)]. To evaluate only the effect of thermal stress on corals, relative *Q*_*m*_ (*ΔQ*_*m*_) was calculated subtracting the control average *Q*_*m control*_ to the treatment average *Q*_*m treatment*_*,* modifying the equation used by Fisher et al.^52^.

### Algal pigment content determinations

Pigment content and symbiont density were determined following the methodology described by Scheufen et al.^9^. Briefly, coral tissue was removed with pressurized air and filtered seawater (0.45 µm). Coral slurry was homogenized and divided into 1 mL aliquots. To determine algal density, one aliquot was preserved adding 200 µL of iodine (Lugol, Sigma) and algal cell counts were performed using a Neubauer chamber. Photosynthetic pigments were extracted in an acetone: DMSO mix (95:5, V:V)^8^. Chlorophyll *a* content was calculated spectrophotometrically using the equations described by Jeffrey and Humphrey^53^. Pigment content and algal density were standardized by coral area, determined by the aluminum foil method^54^.

### Coral optical determinations

To determine variations in coral light absorption capacity, coral estimated absorbance (*D*_*e*_) spectra were calculated from coral reflectance (*R*) measurements as *D*_*e*_ = Log(1/*R*) according to Shibata^55^, Enríquez et al*.*^10^ and Vásquez-Elizondo et al.^38^. Reflectance was measured in each coral fragment every three days. Measurements were performed between 400 and 750 nm using a fiber optic attached to a USB4000 mini spectroradiometer (Ocean Optics Ltd FL), averaging 5 scans per measurement with a boxcar width of 0.4 nm and a resolution of 0.2 nm. Light absorption efficiency by corals was evaluated using the specific absorption coefficient for chlorophyll *a* (a*_Chl*a*_). This coefficient describes the effective area of light collection per mg of Chl *a*, it was calculated using the equation: a*_Chla_ = (*D*_*e675*_/*ρ*)ln (10)^10^, where *D*_*e675*_ is the estimated absorbance at 675 nm and *ρ* is the Chl*a* density of each coral fragment in mg m^−2^.

### Analyses of the optical properties of Ostreobium spp

The presence of *Ostreobium* spp. in *O. faveolata* skeletons was detected from the estimated light absorption spectra. The average absorption spectra of each coral colony was calculated every day of measurement. To determine the presence of *Ostreobium* spp., we calculated the second derivative of the coral absorption spectra, and three main spectral characteristics were evaluated for this analysis: (i) The absorption at 645 nm, which represents the absorbance of chlorophyll *b* (Chl*b*) present only in *Ostreobium* spp.^31,32^; (ii) the absorbance above 700 nm, corresponding to the long-wavelength antenna complexes of *Ostreobium* spp.^31,32^; and (iii) the position of the Chl*a* peak of absorption in the red band^31^ (Fig. 2). In addition, a new index was calculated based on the ratio of the second-derivative analysis of the absorption at 645 relative 680 nm (D_*e645*_/D_*e680*_), to confirm the presence of *Ostreobium* spp. in the skeleton of each coral fragment (Supplementary Fig. S4).

### Numerical model

To further explore the possible effects of *Ostreobium* spp. during coral recovery, we created a numerical model on the software Microsoft^®^ Excel (Version 16.43(200110804) based on: (i) the vertical spectral diffuse attenuation coefficient for downwelling irradiance^56^, (ii) the light absorption spectra of a coral measured in transmittance mode^10^, (iii) the light transmission properties of coral skeletons^38^ and (iv) the in vivo absorption spectra of *Ostreobium* spp. in coral skeletons (Fig. 5). The model simulates light transmission inside coral tissue containing different Chl*a* density as well explains the effect of endolithic algae on the optical properties of coral skeletons and the light environment in hospite.

### Statistical analysis

All results are expressed as mean ± SD. Differences between phenotypes were analyzed using a Student t-test, after testing for assumptions. Two-way ANOVA test and a Post Hoc Tukey tests were using to identify significant differences among days in the control and heat stressed corals. Analyses were conducted using Microsoft^®^ Excel software (Version 16.43(200110804)) and the software SPSS Statistics (Version 28).

## Supplementary Information

Supplementary Information.
