# Supplementary material for: The role of the endolithic alga Ostreobium spp. during coral bleaching recovery
Source: Sci Rep. 2022 Feb 22;12:2977. doi: 10.1038/s41598-022-07017-6 (PMC8863988; doi:10.1038/s41598-022-07017-6)
Supplement: Supplementary file 1 — Supplementary Information. [file 41598_2022_7017_MOESM1_ESM.pdf]

The role of the endolithic alga *Ostreobium* spp. during coral bleaching recovery.

Claudia Tatiana Galindo-Martínez[1,*], Michele Weber[1,2], Viridiana Avila-Magaña[1,3], Susana Enríquez[2], Hiroaki Kitano[4,5], Mónica Medina[1], and Roberto Iglesias-Prieto[1,*]

[1] The Pennsylvania State University, Department of Biology, 208 Mueller Lab, University Park, PA 16802, USA.

[2] Laboratory of Photobiology, Unidad Académica de Sistemas Arrecifales Puerto Morelos, Instituto de Ciencias del Mar y Limnología, Universidad Nacional Autónoma de México (UNAM). Puerto Morelos, Cancún, Q.R., 77580, Mexico.

[3] Current address: Department of Ecology and Evolutionary Biology, University of Colorado, Boulder, USA

[4] Okinawa Institute of Science and Technology Graduate School, Okinawa, Japan.

[5] The Systems Biology Institute, Shinagawa, Tokyo, Japan.

Supplementary Material

Coral and Symbiodinaceae algae genotyping

Total DNA was extracted from ~0.2 grams of coral tissue fine powder obtained from coral fragments by removing coral skeletons on dry ice and liquid nitrogen using a chisel and hammer. DNA was extracted using the Mo Bio Laboratories PowerFecal DNA Isolation Kit, following the manufacturer's standard procedure. Extracts were quantified and diluted to approximately 10 ng/μl. The ITS2 region of the Symbiodiniaceae algal rDNA was amplified using the primers by Pochon et al. [1] with the specific PCR conditions from Avila-Magaña et al.[2]. The PCR products were checked on a 2% agarose gel to make sure they were not over amplified, and we added cycles or re-amplified as necessary to minimize and equalize

amplification for each sample (25-30 cycles). Amplicon products were shipped to the University of Texas sequencing center and sequenced using Illumina MiSeq 250 bp PE libraries, targeting ~50K reads at the Genomic Sequencing and Analysis Facility, University of Texas.

A quality check on the sequencing data was performed using FastQC (v0.11.2). We used bioinformatic pipeline for processing all the samples at once as in Avila-Magaña et al.[2]. In brief, when describing genetic diversity in the amplicons, a single copy of each unique sequence was run through the clustering program USEARCH ([3]). We assumed that 3% divergence represented a new OTU and we performed a blast using the representative sequence from each cluster as a query against the GeoSymBio database ([4]). We defined these references as members of the Symbiodiniaceae community living in the experimental *O. faveolata* fragments. Finally, all reads for each coral fragment were mapped to the reference sequences in order to estimate the symbiont community composition for each fragment by using the usearch_global function. Absolute abundances were normalized by calculating the OTU abundance per coral sample by the total abundance ratio.

Host genotyping was performed by using seven distinct microsatellite loci as described in Baums et al.[5] and Davies et al.[6] (**Table S5**). Briefly, each 20 µl of polymerase chain reaction (PCR) mixture contained 10 ng of DNA template, 0.1 µM fluorescent labeled forward primer, 0.1 µM reverse primer, 0.2 mM dNTP, 1 µl 10X ExTaq buffer, 0.025 U ExTaq Polymerase (Takara Biotechnology) and 0.0125 U Pfu Polymerase (Agilent Technologies). PCR conditions began at 94 °C for 5 min, followed by 35 cycles of 94 °C for 40 s, 60 °C for 2 min, and 72 °C for 60 s and then a 10-min extension period at 72 °C. Amplicons were resolved on agarose gels to verify amplification and molecular weights were analyzed using the ABI 3130XL capillary sequencer with a ROX-labeled size standard.

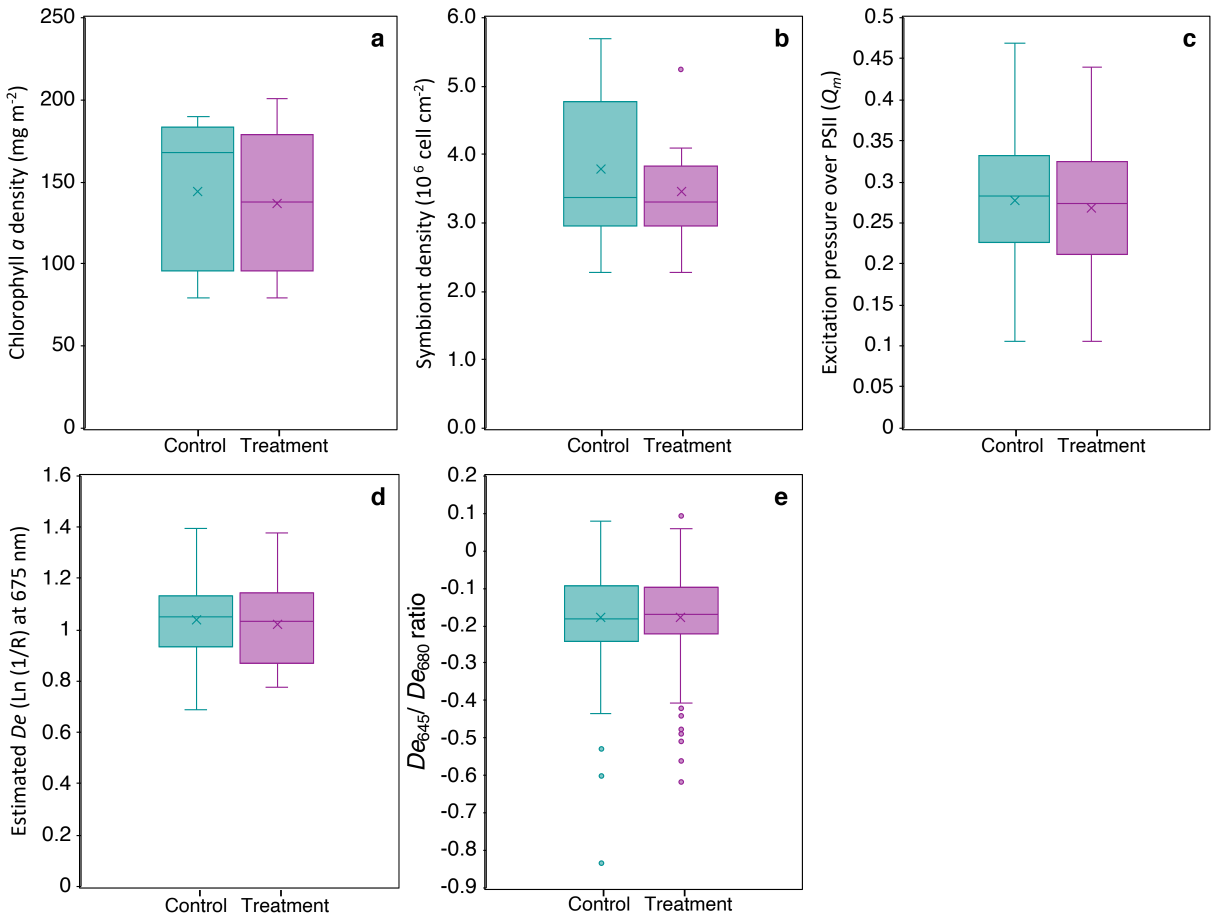

Supplementary figure 1. Characterization of the Control and Treatment coral phenotypes before the B-Re experiments. Box plots for comparison of: **(a)** Chlorophyll *a* density (n= 11 fragments per group); **(b)** symbiont density (n= 11 fragments per group)**; (c)** excitation pressure over PSII (n= 116 fragments per group); **(d)** estimated absorbance at 675 nm, D_{e675} (n= 114 fragments per group), and the **(e)** 645/ 680 ratio of the second-derivative analysis of the absorption spectrum (n= 342 spectra per group) between control (light blue) and heat treated (pink) *Orbicella faveolata* fragments. Boxes encompass the 25 and 75% quartiles of the variation. The central line corresponds to the median, and bars extend to the 95 and 5% of the confidence limits. T-test analysis did not show significant differences among coral groups (T-test, p > 0.05).

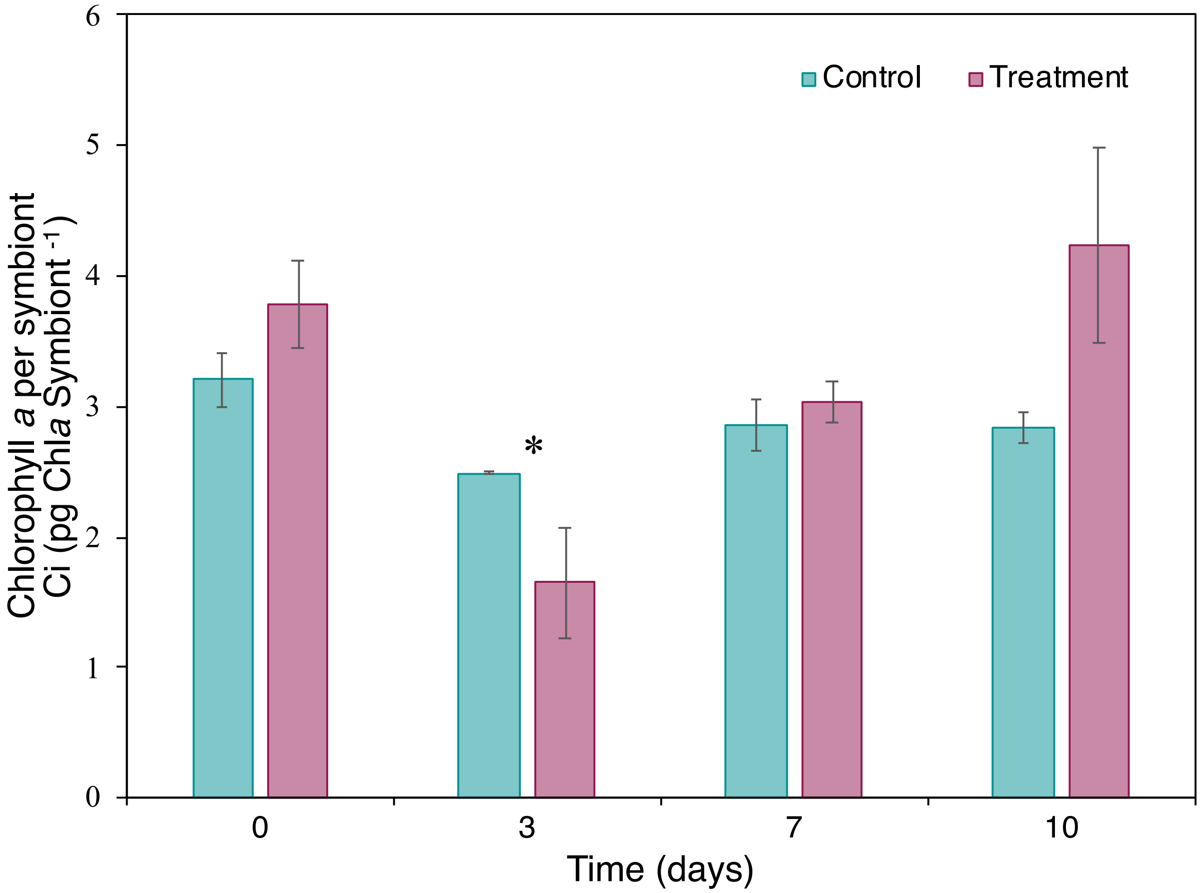

Supplementary figure 2. Variation of the pigment content per symbiont cell (pg $_{Chla}$ cell^{-1}) in *Orbicella faveolata* control (clear blue) and treatment (pink) fragments along the B-Re (n= 3 fragments per group). Values correspond to mean ± SD. Day 0 shows the initial conditions previous the thermal stress. Days 3 to 10 describe the modification during the thermal stress period (32°C). Tukey Post-Hoc test ($p<0.05$) only showed significant differences on day 3 for both control and treatment corals (*).

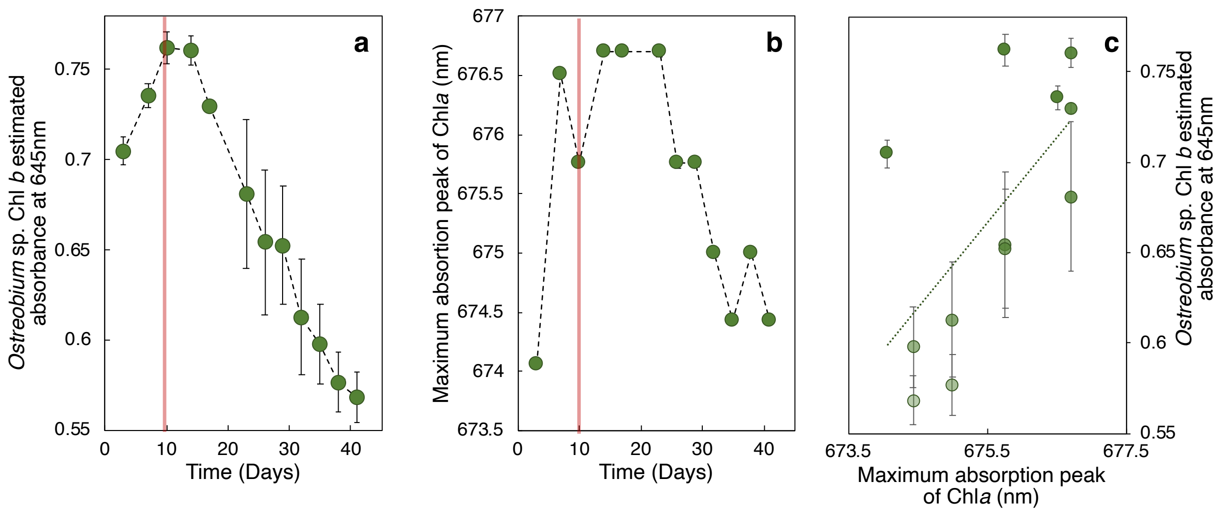

Supplementary figure 3. Description of the spectroscopic parameters in the recovered coral colony 197, **a)** Modification of the estimated absorbance at 645 nm in colony 197 fragments during the B-Re. **b)** Modification of the maximum peak of absorption of chlorophyll a in colony 197 fragments during the B-Re. Red vertical solid line represents the end of the heat stress period. **c)** Modification in the relation between the estimated absorbance at 645 nm and the peak of absorption of chlorophyll *a*. (Fitting function: $De_{645} = 0.047 \pm 0.02^{*} MP_{Chl\ a} - 31.098 \pm 11.71$, $R^2 = 0.42$, $p < 0.05$)

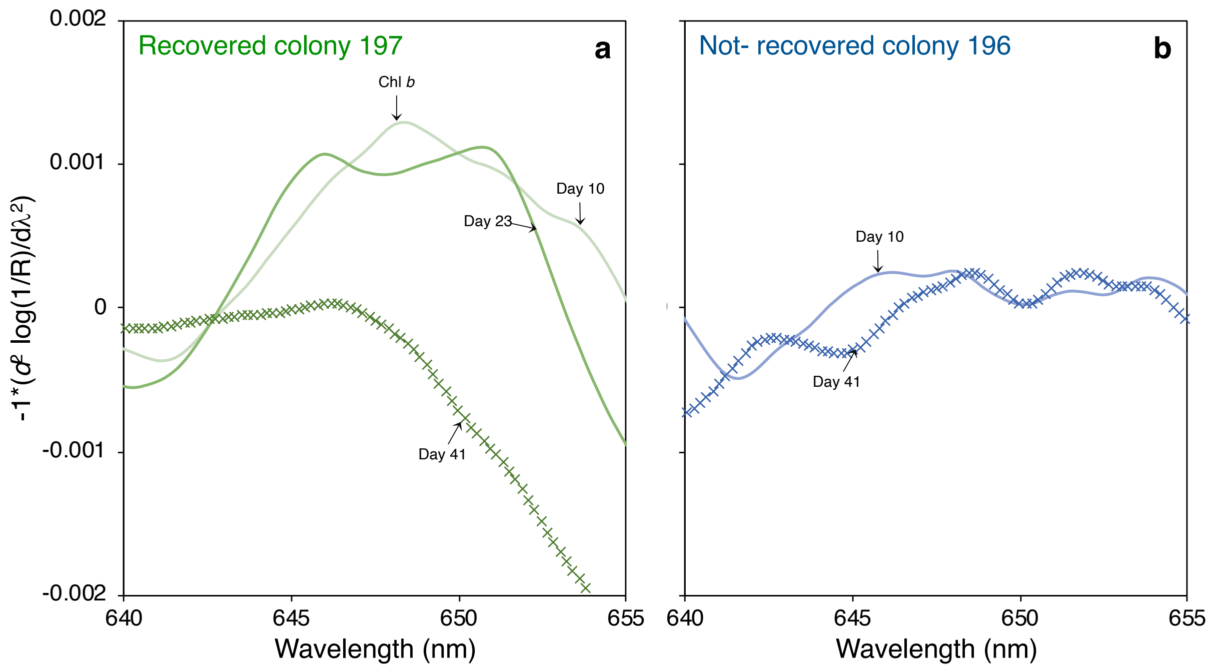

Supplementary figure 4. Detailed comparison of the second-derivative analysis of the absorption spectra in the range of 640 nm to 655 nm in coral fragments from colony 197 **(a)** and coral colony 196 **(b)** on last day of heat stress (32ºC, day 10, light green line and light blue line respectively) and during the recovery period (28ºC, days 23 and 41, dark green line and dark blue line respectively). Symbols in day 41 represents each data in the absorption spectra.

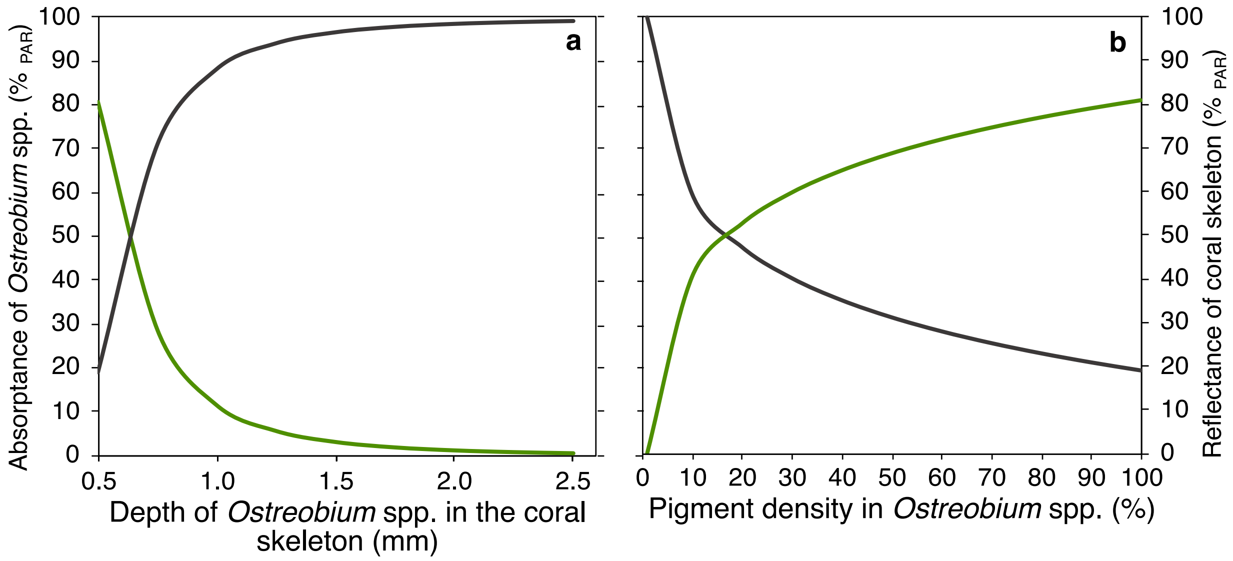

Supplementary figure 5. Simulations of the numerical model indicate the *Ostreobium* spp. effect in the optical properties of the coral skeleton. **a, b)** Modification of the percentage of light absorbed by the *Ostreobium* spp. bloom (green solid line) and the percentage of light reflected by the coral skeleton (gray solid line) as function of the depth of the *Ostreobium* spp. bloom in the coral skeleton **(a)** and the pigment density in *Ostreobium* spp. **(b)**.

TS1. Student t-test analysis comparing the photobiology and spectrophotometric parameters of the *Orbicella faveolata* control and treatment corals on last day of heat stress. Values correspond to the mean ± SD (sample size n). *t*- test (*t*) was conducted after testing of assumptions to determine if differences between groups were statistically significant. Significant values ($p<0.05$) are marked in bold.

Parameters	Control	Treatment	Statistics
Chlorophyll *a* density (mg Chl*a* m^{-2})	135.60 ± 32.83 (10)	11.93 ± 15.95 (11)	**$t_{(13)}$ = 10.79, p < 0.01**
Symbiont density (10^6 cell cm^{-2})	3.84 ± 1.45 (11)	0.28 ± 0.51 (12)	**$t_{(12)}$ = 8.03, p < 0.01**
Excitation pressure over PSII (Q_m) (dimensionless)	0.32 ± 0.10 (116)	0.65 ± 0.08 (116)	**$t_{(223)}$ = -25.25, p < 0.01**
Estimated absorbance (Ln(1/R)) at 675 nm (D_{e675}, dimensionless)	1.02 ± 0.08 (101)	0.36 ± 0.18 (114)	**$t_{(168)}$ = 36.12, p < 0.01**
	**Recovered**	**Non-recovered**	
D_{e645} / D_{e680} ratio (dimensionless)	0.35 ± 0.12 (13)	0.14 ± 0.15 (11)	**$t_{(20)}$ = -3.73, p < 0.01**

TS2. Student t-test analysis comparing the photobiology and spectrophotometric parameters of the *O. faveolata* recovered and non-recovered corals on last day of experiment. Values correspond to the mean ± SD (sample size n). *t*- test (*t*) was conducted after testing of assumptions to determine if differences between groups were statistically significant. Significant values (p<0.05) are marked in bold.

Parameter (units)	Recovered	Non-recovered	Statistics
Excitation pressure over PSII (Q_m) (dimensionless)	0.32 ± 0.12 (25)	0.68 ± 0.23 (23)	**$t_{(37)}$ = 6.67, p < 0.01**
Estimated absorbance (Ln(1/R)) at 675 nm (D_{e675}; dimensionless)	0.72 ± 0.20 (29)	0.22 ± 0.15 (38)	**$t_{(49)}$ = -11.07, p < 0.01**
	**Recovered**	**Control corals (day 10)**	
Estimated absorbance (Ln(1/R)) at 675 nm (D_{e675}; dimensionless)	0.72 ± 0.20 (29)	1.02 ± 0.08 (101)	**$t_{(31)}$ = -7.40, p < 0.01**
	**Not recovered**	**Treatment (day 10)**	
Estimated absorbance (Ln(1/R)) at 675 nm (D_{e675}, dimensionless)	0.22 ± 0.15 (38)	0.36 ± 0.18 (114)	**$t_{(150)}$ = 4.41, p < 0.01**

TS3. Student t-test analysis comparing the photobiology and spectrophotometric parameters of the *O. faveolata* colony 196 and 197 fragments during the B-Re. Values correspond to the mean ± SD (sample size n). *t*- test (*t*) was conducted after testing of assumptions to determine if differences between groups were statistically significant. Significant values (p<0.05) are marked in bold.

Parameter (units)	Control (day 10)	Treatment (day 10)	Statistics
**Coral colony 196**			
Excitation pressure over PSII (Q_m) (dimensionless)	0.26 ± 0.06 (10)	0.61 ± 0.24 (6)	**$t_{(5)}$ = 3.36, p = 0.01**
Estimated absorbance (Ln(1/R)) at 675 nm (D_{e675}, dimensionless)	1.04 ± 0.07 (10)	0.20 ± 0.08 (11)	**$t_{(19)}$ = -23.33, p < 0.01**
**Coral colony 197**			
Excitation pressure over PSII (Q_m) (dimensionless)	0.21 ± 0.09 (9)	0.62 ± 0.35 (6)	**$t_{(5)}$ = 2.79, p = 0.01**
Estimated absorbance (Ln(1/R)) at 675 nm (D_{e675}, dimensionless)	1.01 ± 0.05 (9)	0.25 ± 0.11 (13)	**$t_{(19)}$ = -21.32, p < 0.01**
	**Treatment (day 10)**	**Treatment (day 40)**	
**Coral colony 196**			
Excitation pressure over PSII (Q_m) (dimensionless)	0.61 ± 0.24 (6)	0.56 ± 0.26 (6)	$t_{(9)}$ = 0.35, p > 0.05
Estimated absorbance (Ln(1/R)) at 675 nm (D_{e675}, dimensionless)	0.20 ± 0.08 (11)	0.16 ± 0.08 (9)	$t_{(17)}$ = 1.14, p > 0.05
**Coral colony 197**			
Excitation pressure over PSII (Q_m) (dimensionless)	0.62 ± 0.35 (6)	0.20 ± 0.12 (3)	**$t_{(7)}$ = 2.68, p < 0.05**
Estimated absorbance (Ln(1/R)) at 675 nm (D_{e675}, dimensionless)	0.25 ± 0.11 (13)	0.71 ± 0.15 (9)	**$t_{(14)}$ = -8.02, p < 0.01**

TS4. Detailed information for each *Orbicella faveolata* colony analyzed during this research. Colony number, initial and final number of fragments (n), coral host genotype and symbiont genotype are indicated for each colony. Differences in the number of coral fragments between the initial and final time are due to sampling of coral fragments for analysis during the experiment.

**Coral colony number**	**Initial fragments (n) (Total, T; Control, C; Treatment, H)**	**Final fragments (n) (Control, C; Recovered, R; Not-recovered: NR)**	**Coral genotype**	**Dominant algal symbiont**
196	T: 37<br>C: 19<br>H: 18	C: 8<br>R: 0<br>NR: 9	Orb1512	*Cladocopium* sp
197	T: 37<br>C: 19<br>H: 18	C: 5<br>R: 9<br>NR: 0	Orb1512	*Cladocopium* sp
198	T: 41<br>C: 21<br>H: 20	C: 10<br>R: 4<br>NR: 7	Orb1514	*Cladocopium* sp
775	T: 18<br>C: 9<br>H: 9	C: 2<br>R: 3<br>NR: 0	nd	nd
765	T: 22<br>C: 11<br>H: 11	C: 5<br>R: 6<br>NR: 0	nd	nd
766	T: 23<br>C: 11<br>H: 12	C: 3<br>R: 5<br>NR: 0	nd	nd
191	T: 36<br>C: 16<br>H: 18	C: 8<br>R: 0<br>NR: 9	nd	nd
192	T: 26<br>C: 13<br>H: 13	C: 5<br>R: 0<br>NR: 4	nd	nd
193	T: 23<br>C: 11<br>H: 12	C: 5<br>R: 0<br>NR: 6	nd	nd
194	T: 24<br>C: 12<br>H: 12	C: 4<br>R: 0<br>NR: 5	nd	nd
191´	T: 15<br>C: 7<br>H: 8	C: 2<br>R: 3<br>NR: 0	nd	nd
193´	T: 16<br>C: 8<br>H: 8	C: 2<br>R: 3<br>NR: 0	nd	nd

194´	T: 26<br>C: 13<br>H: 13	C: 7<br>R: 0<br>NR: 8	nd	nd

TS5. Simple sequence repeats (SSR) markers used to genotype *Orbicella faveolata* [[6,7]]

Locus (Repeat)	Primer sequence [a] 5´-3´	Accession no.	Observed (bp)
maMS11	F: NED-CAGACGGATTAAATAGTCTCCCA<br>R: GACGAATTTTGCCGAGTCAC	AY395775	308–364
maMS2-8	F: 6-FAM-CCCCTTTGTCACACATCTTTC<br>R: ATGAAGGATAGGCCGCACT	AY395779	175–229
Mfav4	F: FAM-ATTAGGGTCGAGGTCAAGGG<br>R: CAAGCCCGAATAAAGCATA	GW254471	368-416
Mfav8	F: NED-GACCCCAGTTCAACTCCTCA<br>R: TCCTGTCCCTCTTGTATGCC	GW250389	288-357
Mfav5	F: FAM-AATGCATTTCCCTGCTATCG<br>R: TGCACGACCCTGTACAACAT	GW257722	342-394
Mfav7	F: HEX-GCCGAATCCGTCTTTTGATA<br>R: GCTGAGGTTGCTCTTGCTGT	GW248775	459-519
Mfav9	F: NED-ATTCGTGACAAAACGAGGGTC<br>R: AATTGACCGAAAAGTGACCG	GW249713	276-322

[a] FAM, HEX and NED indicate the fluorescent primer

Supplementary references

1. Pochon, X., Pawlowski, J., Zaninetti, L. & Rowan, R. High genetic diversity and relative specificity among *Symbiodinium* -like endosymbiotic dinoflagellates in soritid foraminiferans. *Marine Biology* **139**, 1069–1078 (2001).
2. Avila-Magaña, V. *et al.* Elucidating gene expression adaptation of phylogenetically divergent coral holobionts under heat stress. *Nat Commun* **12**, 1–16 (2021).
3. Edgar, R. C. Search and clustering orders of magnitude faster than BLAST. *Bioinformatics* **26**, 2460–2461 (2010).
4. Franklin, E. C., Stat, M., Pochon, X., Putnam, H. M. & Gates, R. D. GeoSymbio: a hybrid, cloud-based web application of global geospatial bioinformatics and ecoinformatics for *Symbiodinium*-host symbioses. *Molecular Ecology Resources* **12**, 369–373 (2012).
5. Baums, I. B., Johnson, M. E., Devlin-Durante, M. K. & Miller, M. W. Host population genetic structure and zooxanthellae diversity of two reef-building coral species along the Florida Reef Tract and wider Caribbean. *Coral Reefs* **29**, 835–842 (2010).
6. Davies, S. W. *et al.* Novel polymorphic microsatellite markers for population genetics of the endangered Caribbean star coral, *Montastraea faveolata*. *Mar Biodiv* **43**, 167–172 (2013).
7. Foster, N. L., Baums, I. B. & Mumby, P. J. Sexual vs. asexual reproduction in an ecosystem engineer: the massive coral *Montastraea annularis*. *Journal of Animal Ecology* **76**, 384–391 (2007).
